# A phase I study assessing the safety, tolerability, immunogenicity, and low-density lipoprotein cholesterol-lowering activity of immunotherapeutics targeting PCSK9 

**DOI:** 10.1007/s00228-021-03149-2

**Published:** 2021-05-10

**Authors:** Markus Zeitlinger, Martin Bauer, Roman Reindl-Schwaighofer, Robert M. Stoekenbroek, Gilles Lambert, Evelyn Berger-Sieczkowski, Heimo Lagler, Zoe Oesterreicher, Beatrix Wulkersdorfer, Petra Lührs, Gergana Galabova, Carsten Schwenke, Robert M. Mader, Rossella Medori, Christine Landlinger, Alexandra Kutzelnigg, Günther Staffler

**Affiliations:** 1 Department of Clinical Pharmacology, Medical University of Vienna, Währinger Gürtel 18-20, 1090 Vienna, Austria; 2 Department of Vascular Surgery, Academic Medical Center, University of Amsterdam, P.O. Box 22660, 1100 DD Amsterdam, Netherlands; 3 Laboratoire Inserm, UMR 1188 DéTROI, Université de La Réunion, 2 Rue Maxime Rivière, 97490 Sainte Clotilde, France; 4 Department of Neurology, Medical University of Vienna, Währinger Gürtel 18-20, 1090 Vienna, Austria; 5 Department of Medicine I, Medical University of Vienna, Währinger Gürtel 18-20, 1090 Vienna, Austria; 6 AFFiRiS AG, Karl Farkas Gasse 22, 1030 Vienna, Austria; 7 Origenis GmbH, Am Klopferspitz 19a, 82152 Martinsried, Germany; 8 SCO:SSiS, Karmeliterweg 42, 13465 Berlin, Germany

**Keywords:** Hypercholesterolemia, PCSK9, Active immunotherapy, In vivo antibody development, LDLc reduction, First-in-human study

## Abstract

**Purpose:**

AT04A and AT06A are two AFFITOPE® peptide vaccine candidates being developed for the treatment of hypercholesterolemia by inducing proprotein convertase subtilisin/kexin type 9 (PCSK9)-specific antibodies. This study aimed to investigate safety, tolerability, antibody development, and reduction of low-density lipoprotein cholesterol (LDLc) following four subcutaneous immunizations.

**Methods:**

This phase I, single-blind, randomized, placebo-controlled study was conducted in a total of 72 healthy subjects with a mean fasting LDLc level at baseline of 117.1 mg/dL (range 77–196 mg/dL). Each cohort enrolled 24 subjects to receive three priming immunizations at weeks 0, 4, and 8 and to receive a single booster immunization at week 60 of either AT04A, AT06A, or placebo. In addition to safety (primary objective), the antigenic peptide- and PCSK9-specific antibody response and the impact on LDLc were evaluated over a period of 90 weeks.

**Results:**

The most common systemic treatment-related adverse events (AEs) reported were fatigue, headache, and myalgia in 75% of subjects in the AT06A group and 58% and 46% of subjects in the placebo and AT04A groups, respectively. Injection site reactions (ISR) representing 63% of all treatment-emergent adverse events (TEAEs), were transient and mostly of mild or moderate intensity and rarely severe (3%). Both active treatments triggered a robust, long-lasting antibody response towards the antigenic peptides used for immunization that optimally cross-reacted with the target epitope on PCSK9. In the AT04A group, a reduction in serum LDLc was observed with a mean peak reduction of 11.2% and 13.3% from baseline compared to placebo at week 20 and 70 respectively, and over the whole study period, the mean LDLc reduction for the AT04A group vs. placebo was −7.2% (95% CI [−10.4 to −3.9], *P* < 0.0001). In this group, PCSK9 target epitope titers above 50 were associated with clinically relevant LDLc reductions with an individual maximal decrease of 39%.

**Conclusions:**

Although both AT04A and AT06 were safe and immunogenic, only AT04A demonstrated significant LDLc-lowering activity, justifying further development.

**Trial registration:**

EudraCT: 2015-001719-11. ClinicalTrials.gov Identifier: NCT02508896.

**Supplementary Information:**

The online version contains supplementary material available at 10.1007/s00228-021-03149-2.

## Introduction

Cardiovascular disease is responsible for 18 million deaths annually [1], with complications including heart attack, stroke, and peripheral artery occlusive disease [1]. Increased levels of lipids (hyperlipidaemia), particularly low-density lipoprotein cholesterol (LDLc) is considered the major risk factor for developing atherosclerotic plaques, causing a variety of cardiovascular events [2].

Cardiovascular risk has been addressed by reducing cholesterol with medication, particularly statins, alone or in combination with ezetimibe. However, statins are often insufficient to achieve guideline-recommended LDLc levels [3]. Side effects and poor adherence to statin therapy are important factors that prevent realization of the benefits of statins in routine care [4, 5]. Other approved treatment alternatives include monoclonal antibodies targeting PCSK9 (proprotein convertase subtilisin/kexin type 9), a central regulator of the LDL receptor (LDLR) [6, 7]. PCSK9 binds and downregulates LDLR expressed on hepatocytes [8], with a decrease in active circulating PCSK9 LDLR density increases, which in turn increases LDL uptake from circulation and reduces serum LDLc levels [9]. PCSK9 inhibition through passive immunization (i.e., monoclonal antibodies) has proven to be successful but is costly, requiring self-administration every 2 or 4 weeks [10–15].

However, atherosclerosis is a chronic disease requiring continued treatment, raising issues over time with patient compliance, side effects, and cost. Current therapies being investigated to reduce the frequency of treatment include siRNA-based therapies such as inclisiran [16, 17], with proposed administration twice yearly.

The aim of specific active immunotherapy (SAIT) is to provide long-lasting PCSK9 inhibition through the activation of the body’s own immune system, to produce an antibody response following priming immunizations and one booster immunization per year. The core element of the SAIT technology involves immunization with short peptides (AFFITOPE®s) with a sequence differing from the native sequence but mimicking (parts of) the native sequence of the target protein. As selected candidates do not exhibit sequence identity with other human proteins, they are “foreign” to the human immune system. This endows the technology with a conceptual advantage. Immune responses are more readily elicited toward foreign- as compared to self-proteins as the organism protects “self” from being destructed by an immune attack via “central” and “peripheral” tolerance mechanisms. The SAIT candidates AT04 and AT06 have been designed with the aim of breaking tolerance against PCSK9 protein, of inducing a specific oligoclonal antibody response that cross-reacts and inhibits the physiological target protein PCSK9, without induction of auto-immunity.

In preclinical models, SAIT against PCSK9 induced high, persistent antibody levels against PCSK9, causing a significant reduction in LDLc [18, 19], consequently reducing the development of atherosclerotic lesions [19]. In all preclinical experiments as well as in the toxicology studies, both SAIT products have been demonstrated to be safe and well tolerated.

This first-in-human trial assessed the safety, tolerability, immunogenicity, and LDLc-lowering activity of two PCSK9-targeting candidates, AT04A and AT06A, in healthy volunteers.

## Methods

### Subjects

Seventy-two adult male and female healthy subjects (18 to 65 years) were enrolled in the study, which was conducted from 22 July 2015 to 31 August 2017 at a single center in Vienna, Austria. At screening, a fasting LDLc ≥ 75 to ≤ 200 mg/dl, fasting triglycerides ≤ 400 mg/dl, and a BMI between 19 and 35 were required for inclusion. Females of childbearing potential were eligible if they accepted use of contraception. Exclusion criteria included history of autoimmune or severe allergic diseases and prior and/or current treatment with immune modulating drugs and treatment with medication known to influence high-density lipoprotein cholesterol (HDLc), LDLc, and total cholesterol concentrations up to 6 weeks prior to screening. Subjects were instructed to report lifestyle changes during the study (e.g., exercise, attempting weight loss, smoking status).

### Study design and treatment

This phase I study was a randomized, parallel group, single-blind, single-center trial to assess the safety and tolerability (primary study objective) of two AFFITOPE® SAIT candidates, AT04A and AT06A, in comparison to placebo in healthy subjects. The secondary objectives were to analyze the immune response and the LDLc-lowering activity of treatment.

AT04A and AT06A consist of 10 amino acid long peptide variants of the epitope PCSK9 aa 153–162, with one and two amino acid substitutions, respectively, coupled to the carrier protein KLH and adjuvanted with aluminum hydroxide (Alhydrogel® – 0.5 mg aluminum equivalent per dose) in phosphate-buffered saline (PBS). Placebo contained 0.5 mg aluminum hydroxide in 0.5 mL PBS.

**Fig. 1:**
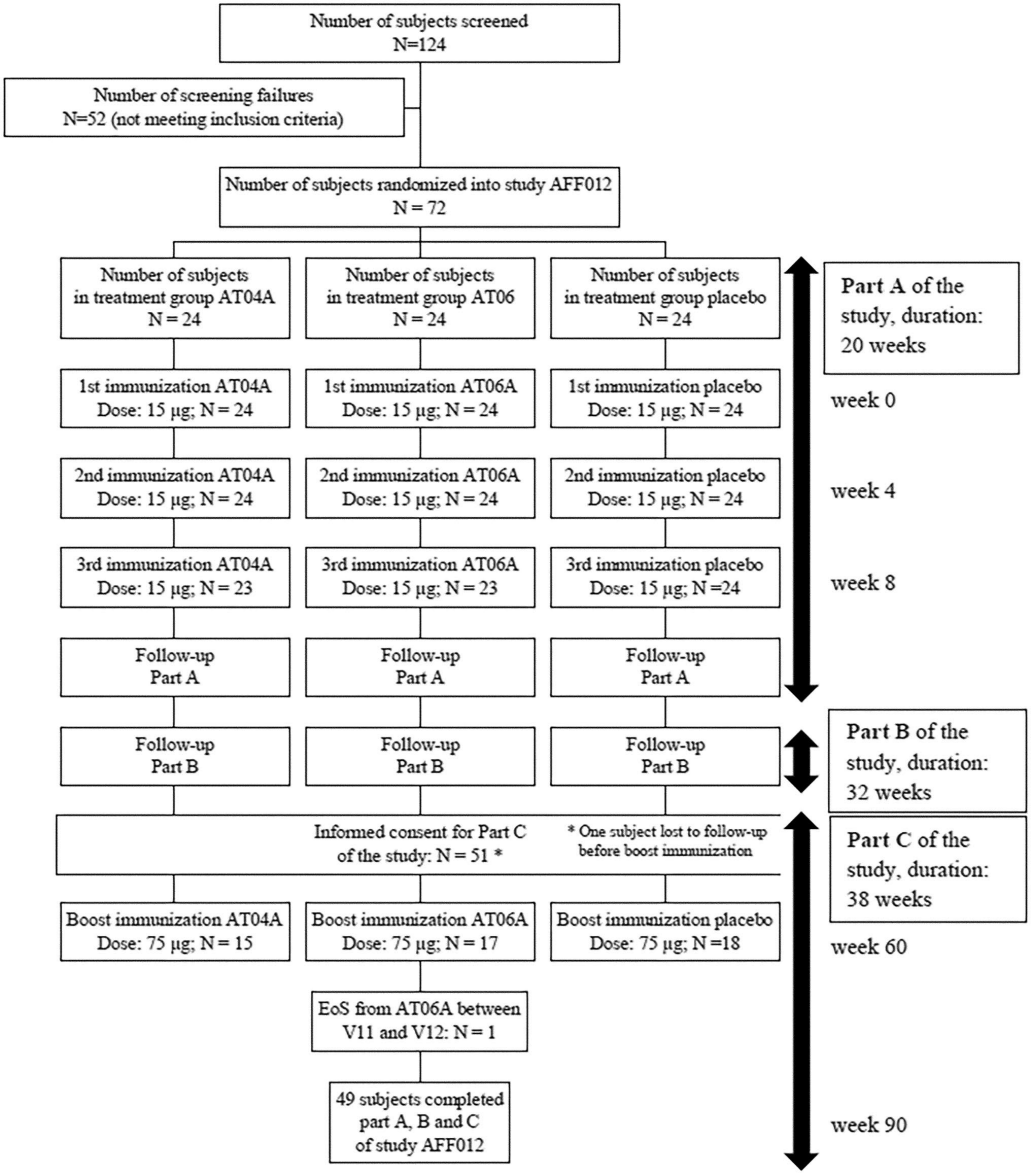
Study design

Informed consent was obtained and participants’ eligibility assessed based on inclusion/exclusion criteria. The study was divided into three parts (Fig. 1). Part A (eight visits) covered three subcutaneous immunizations (weeks 0, 4, 8), with a dose of 15 μg AFFITOPE®, with visits 2 weeks after each immunization and follow-up to week 20. The randomization code was generated according to the permuted blocks method with fixed block size, with subjects randomized 1:1:1 to AT04A, AT06A, or placebo treatment. In Part B, two safety follow-up visits were performed without treatment. In Part C (seven visits), participants were offered a single booster immunization at a five-fold higher dose (75 μg AFFITOPE®), administered at week 60, with five subsequent follow-up visits and the final visit at week 90. Doses correspond to the net antigen AT04 or AT06 peptide amount of the applied drug product.

### Safety assessments

Safety examinations included repeated assessments of systemic and local AEs, periodic physical examinations and electrocardiograms (ECGs), monitoring of vital signs, and clinical laboratory assays (haematology, coagulation, urinalysis, clinical chemistry including C-reactive protein as sensitive parameter for inflammation and liver enzymes, and lipid metabolism).

Peripheral mononuclear blood cells (PBMCs) for analysis of T cell activation were drawn at baseline, three weeks after the priming immunization schedule and before and six weeks after the booster.

### Assessment of antibody development and LDLc lowering

Venous blood was collected before and following immunizations at weeks 0, 2, 6, 10, 20, 39, 52, 60, 62, 66, 70, 74, 82, and 90. Serum samples were serially diluted (1:3 dilution steps) and antibody titers against the AT04 and AT06 immunizing peptides, and against the native epitope on the target protein PCSK9 were assessed by an external provider (eBioscience, Vienna) using an ELISA validated to specifically detect IgG antibodies in sera. Titers were expressed as the serum dilution giving half-maximal binding. The specificity of the signal was confirmed by assaying pre-immune sera, which gave signals below background (< 10). For serum antibody concentration determination, calibration free concentration analysis (CFCA) was performed using surface plasmon resonance (Biacore T200).

A second ELISA was used for serum PCSK9 concentration determination, measuring the total protein level of PCSK9 and to detect free PCSK9 (not bound to antibodies induced by AT04A or AT06A). In order to evaluate the clinical activity of the treatment, concentrations of total cholesterol, high-density lipoprotein (HDL) cholesterol and triglycerides were determined at the same time points as done for antibody titer determinations by the central laboratory of the Medical University of Vienna and LDLc was calculated using the Friedewald Equation ([LDL-chol] = [total chol] − [HDL-chol] − [TG]/5). LDLc differences were calculated as relative changes over time with the baseline of each subject set to 100%.

### Statistical analysis

Primary statistical analyses were performed based on the three treatment groups (univariate and multivariate statistics). Statistical tests used were descriptive, hypothesis-generating, and performed two-sided. The sample size was based on a clinical rationale in the absence of prior in-human data to base a sample size calculation on. No formal hypothesis testing was performed, as this was an exploratory study.

Differences between two independent groups were tested by the Mann–Whitney test for metric and ordinal data, and by Fisher’s exact test for categorical data. For the comparison of multiple samples, nonparametric Kruskal–Wallis test was used, followed by pairwise comparison of the treatment groups using the Mann–Whitney test (for significant differences). For matched pairs of samples, e.g., differences before/after treatment, the Wilcoxon sign rank test was applied.

To assess individual profiles of immunological response and selected lipid parameters, several post hoc analyses were performed, using a mixed linear model with the factors treatment, visit, treatment × visit, and baseline analyzed as absolute and relative changes considering antibody titers and cholesterol-related parameters including triglycerides. The full analysis set was used, including 48 subjects who received all immunizations (per protocol) with available lipid parameters until the study end. Analyses were performed with STATA Version 14.2 (StataCorp. 2015, Release 14) and SAS 9.4 (SAS Institute Inc.). Figures were prepared with R [20].

## Results

### Demographics and patient disposition

In total, 124 subjects were screened, 72 enrolled (Table 1), and 70 subjects completed Parts A and B. Two subjects on active treatment did not receive the third immunization, due to severe injection site reactions (ISR) after the second immunization. Fifty subjects received the booster immunization in study Part C, with 49 subjects completing the study (Fig. 1).

**Table 1:** Subject demographics at baseline

Parameter		AT04A (*n* = 24)	AT06A (*n* = 24)	Placebo (*n* = 24)	Total (*n* = 72)
Age	Mean in years (range)	41.1 (21–57)	44.0 (21–62)	43.3 (21–61)	42.8 (21–62)
Sex	(f/m)	14 f/10 m	16 f/8 m	12 f/12 m	42 f/30 m
BMI	Mean (range)	24.6 (20–31)	25.9 (21–32)	24.3 (20–29)	24.9 (20–32)
LDLc	Mean in mg/dL (range)	121.2 (84–196)	111.0 (77–165)	119.0 (83–170)	117.1 (77–196)
HDLc	Mean in mg/dL (range)	61.0 (37–88)	66.6 (40–107)	63.1 (33–106)	63.6 (33–197)
TC	Mean in mg/dL (range)	201.9 (142–294)	195.8 (156–252)	203.3 (158–286)	200.3 (142–294)

*AT04A* AFFITOPE® AT04A conjugate, *AT06A* AFFITOPE® AT06A conjugate, *f* female, *m* male, *BMI* body mass index, *LDLc* low-density lipoprotein cholesterol, *HDLc* high-density lipoprotein cholesterol, *TC* total cholesterol

### Safety

Both immunotherapeutics were safe and well tolerated, with no deaths, no treatment-related SAEs (Table 2, Supplementary Table 1) and no subjects withdrawn due to TEAEs (any AE occurring after the first treatment). Among 72 enrolled subjects, 67 (93.1%) experienced at least one systemic TEAE and 71 (98.6%) subjects experienced ISRs.

**Table 2:** Summary of adverse events per treatment group

	Study treatment:		
	AT04A	AT06A	Placebo
	*n *(%)	*n* (%)	*n* (%)
No. of subjects who died	0 (0%)	0 (0%)	0 (0%)
No. of subjects with SAEs	2 (8%)	1 (4%)	2(8%)
No. of subjects with related SAEs	0 (0%)	0 (0%)	0 (0%)
No. of subjects discontinued due to TEAEs	0 (0%)	0 (0%)	0 (0%)
No. of subjects reporting any AE	24 (100%)	24 (100%)	23 (96%)
No. of subjects with systemic TEAEs	22 (92%)	23 (96%)	22 (92%)
No. of subjects with related systemic TEAEs	11 (46%)	18 (75%)	14 (58%)
No. of subjects with mild related systemic TEAEs	10 (42%)	18 (75%)	14 (58%)
No. of subjects with moderate related systemic TEAEs	5 (21%)	5 (21%)	2 (8%)
No. of subjects with severe related systemic TEAEs	0 (0%)	1 (4%)	0 (0%)
No. of subjects with headache	10 (42%)	16 (67%)	13 (54%)
No. of subjects with fatigue	9 (38%)	14 (58%)	9 (38%)
No. of subjects with myalgia	6 (25%)	9 (38%)	8 (33%)
No. of subjects with ISRs	24 (100%)	24 (100%)	23 (96%)
No. of subjects with mild ISR after 1st vaccination	22 (92%)	23 (96%)	22 (92%)
No. of subjects with moderate ISR after 1st vaccination	9 (38%)	11 (46%)	1 (4%)
No. of subjects with severe ISR after 1st vaccination	0 (0%)	2 (8%)	0 (0%)
No. of subjects with mild ISR after 2nd vaccination	23 (96%)	21 (88%)	21 (88%)
No. of subjects with moderate ISR after 2nd vaccination	10 (42%)	13 (54%)	5 (21%)
No. of subjects with severe ISR after 2nd vaccination	1 (4%)	1 (4%)	0 (0%)
No. of subjects with mild ISR after 3rd vaccination	22 (96%)	20 (87%)	21 (88%)
No. of subjects with moderate ISR after 3rd vaccination	9 (39%)	8 (35%)	2 (8%)
No. of subjects with severe ISR after 3rd vaccination	0 (0%)	1 (4%)	0 (0%)
No. of subjects with mild ISR after the booster	11 (73%)	16 (94%)	13 (72%)
No. of subjects with moderate ISR after the booster	9 (60%)	9 (53%)	1 (6%)
No. of subjects with severe ISR after the booster	4 (27%)	2 (12%)	0 (0%)
No. of subjects with erythema	22 (92%)	24 (100%)	23 (96%)
No. of subjects with induration	23 (96%)	23 (96%)	17 (71%)
No. of subjects with swelling	21 (88%)	23 (96%)	18 (75%)
No. of subjects with granuloma	22 (92%)	23 (96%)	10 (42%)
No. of subjects with pain	20 (83%)	17 (71%)	20 (83%)

*n* number of subjects, (%) percentage of total subjects in each group. *n *= 24 throughout, except after the 3rd vaccination where the subject numbers were 23, 23, and 24 for groups AT04A, AT06A, and placebo, respectively, and after the booster where the subject numbers were 15, 17, and 18, respectively (as described in Fig. 1). Severity of ISRs was rated according to the diameter of redness, swelling, and hardening at the subcutaneous injection site with a diameter > 10 cm qualifying for severe reaction

*AE *adverse event, *SAE* serious AE, *TEAE* treatment emergent AE, *ISR* injection site reaction

Related systemic TEAEs were experienced by 46% (AT04A), 75% (AT06A), and 58% (placebo) of subjects. However, the majority of systemic TEAEs were of mild or moderate intensity, with only one severe systemic TEAE classified as probably related to immunization (AT06A), comprising a transient episode of asthma rapidly controlled with inhalation of fenoterol/ipratropium bromide (Supplementary Tables 2 and 3). The most commonly experienced systemic TEAEs were headache, fatigue, and myalgia.

Injection site reactions (ISRs) accounted for 63% of the recorded AEs classified as related to study treatment, occurring more frequently in active treatment groups (Table 2). Erythema, induration, swelling, granuloma, and pain were reported most frequently, mostly mild or moderate in intensity. Severe ISRs were reported in 8 (16.7%) subjects, most were transient, three (6.3%) required short-term medication. The frequency of injection site reactions was constant over time during the three priming immunizations. However, the number of subjects with severe ISRs increased after the booster with AT04, but not AT06.

Baseline safety parameters were within physiological ranges and were similar across treatment groups. There were no clinically significant changes over time in vital signs, ECG, hematology, coagulation, clinical chemistry of immune parameters (including the absence of complement activation, or an increase in circulating immune complexes) in both treatment groups relative to placebo.

Regular analyses of PMBCs obtained at baseline, following the priming immunizations and until six weeks after the booster immunization, ruled out a systemic activation of cytotoxic T cells specific for PCSK9.

### Both SAIT candidates induced a strong PCSK9-reactive antibody response

**Fig. 2:**
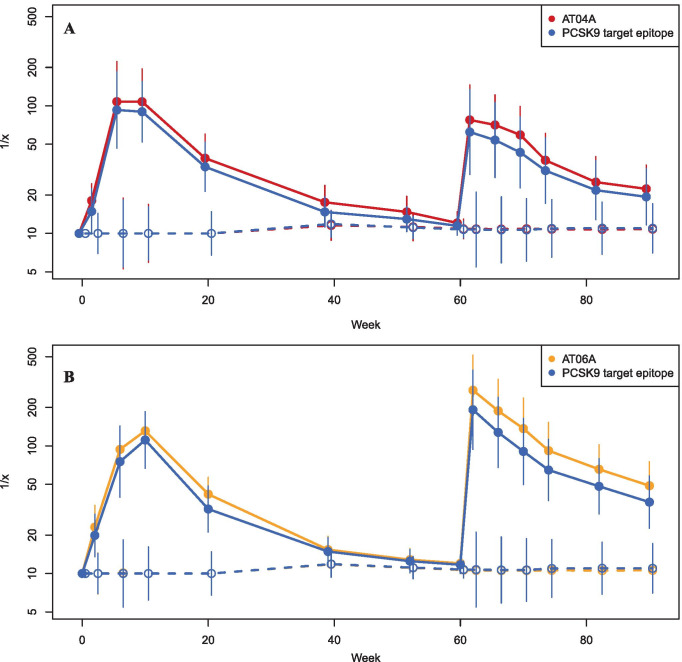
Titers against immunizing peptides AT04 and AT06, and the PCSK9 target epitope over time

Both AT04A (Fig. 2a) and AT06A (Fig. 2b), induced a strong and long-lasting humoral immune response (isotype pattern containing IgG1, IgG3 and IgG4) against the immunizing peptides AT04 and AT06. The geometric mean of the half-max titer increased after two immunizations (i.e., within 6 weeks) from baseline (titer ≤ 1:10) to 1:159 and 1:101 in the AT04A and AT06A group, respectively, with values after the third injection (week 10) only slightly higher at 1:169 and 1:159, respectively. Titers declined over time with an elimination half-life of approximately 12 weeks, reaching baseline levels at week 60. Booster immunization reactivated a strong antibody response with rapid onset in both treatment groups (Fig. 2).

Anti-PCSK9 epitope geometric mean titers increased after three immunizations from baseline (≤ 1:10) to 1:134 and 1:125 in the AT04A and AT06A group respectively, and were marginally below the titers against the immunizing peptides, indicating strong cross-reactivity of treatment-induced antibodies to the target. Titers against the native PCSK9 target epitope displayed a time profile very similar to that observed with AT04 and AT06 (Fig. 2a, b). Seroconversion, defined as a fourfold increase of titers over baseline, was obtained in 21 (87.5%) and 23 (95.8%) subjects in the AT04A and AT06A groups, respectively. Placebo-immunized subjects exhibited no immune response against AT04, AT06 or the PCSK9 target epitope (Fig. 2). The concentration of IgG antibodies against the immunizing peptides was analyzed by CFCA using surface plasmon resonance. Group mean antibody levels reached serum concentrations around 1–2 µg/mL 2 weeks after the second immunization (week 6) and 2 weeks (week 62) after the booster (data not shown).

No differences in total and free PCSK9 concentrations between groups were detected during the study (Supplementary Tables 4 and 5).

### Impact of the immunizations on lipid parameters

Analysis of the relative change of lipid parameters from baseline was performed post hoc, based on the 48 subjects who received the booster immunization and completed the study per protocol, with one subject excluded (prohibited concomitant medication, atorvastatin).

**Fig. 3:**
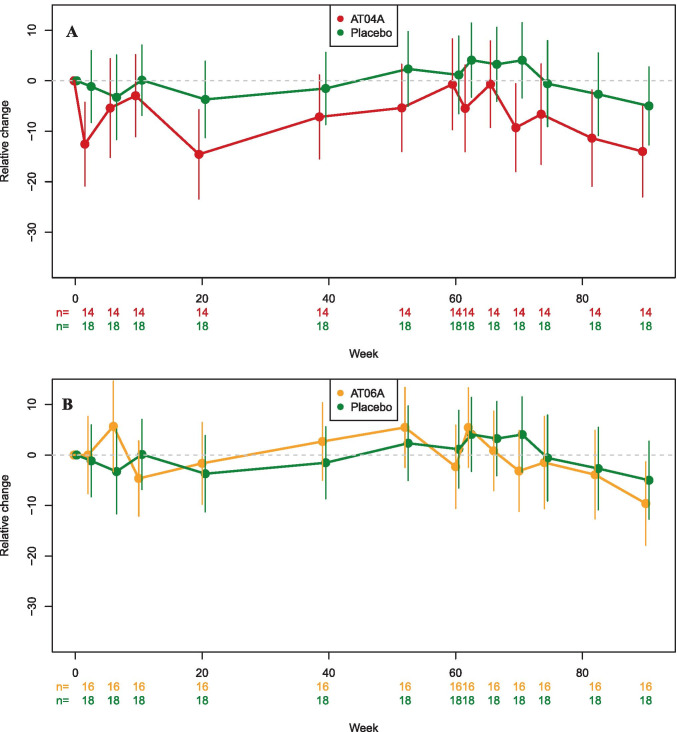
Impact of the active treatments on LDL cholesterol (post hoc analysis)

**Fig. 4:**
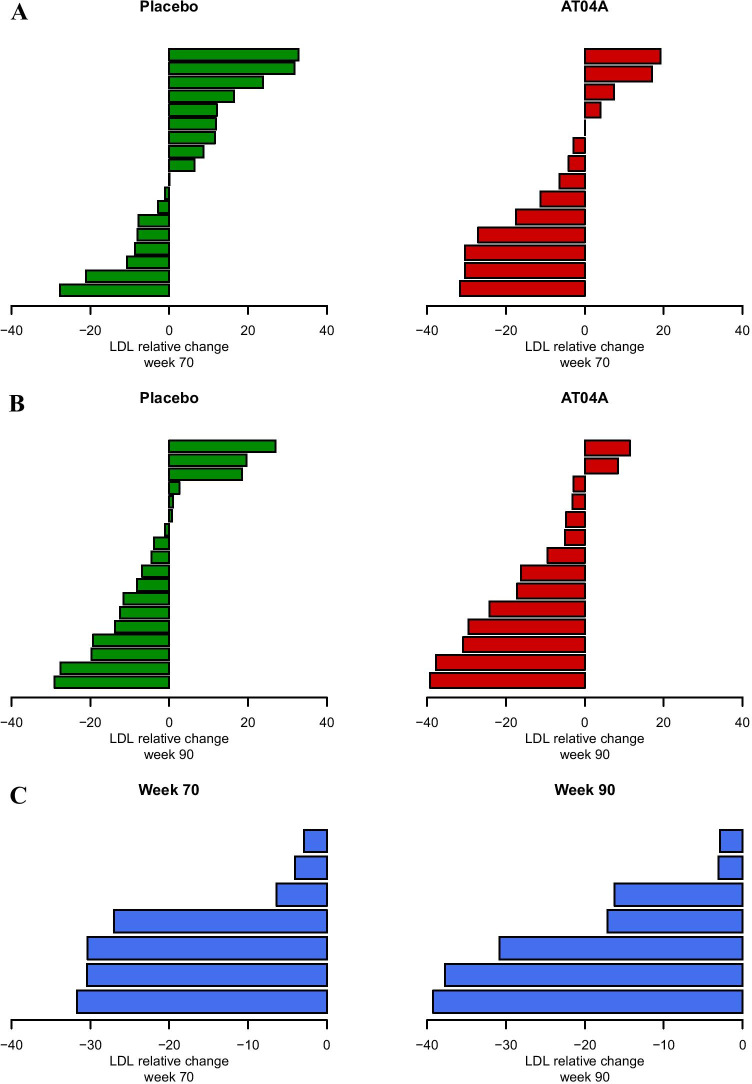
Waterfall plot for the individual LDLc change upon exposure to AFFITOPE® AT04A at week 70 and week 90

The immune response against the PCSK9 target epitope was equally high in both treatment groups, however, the effect on lipid metabolism was more pronounced in AT04A-immunized subjects, with a mean peak reduction in serum LDLc of 11.2% and 13.3% from baseline at weeks 20 and 70, respectively, compared to placebo. Also, a statistically significant reduction of LDLc over the whole study period of 90 weeks was observed in the AT04A group with a mean reduction of 7.2% (*P* < 0.0001). In contrast, no significant difference to placebo was observed for the AT06A group (Fig. 3b, Supplementary Table 6). The relative change in LDLc values from baseline in subjects of the AT04A (*n* = 14) and the placebo (*n* = 18) groups at week 70 (interim analysis) and week 90 (final analysis) are illustrated in Fig. 4. As expected, the individuals in the placebo group showing an increase or decrease of LDLc levels compared to baseline are equally distributed. In the AT04A treated group, however, 9 and 12 out of 14 subjects at week 70 and 90, respectively, showed LDLc lowering (Fig. 4a, b).

All subjects in the AT04A group demonstrating a strong immune response, with a PCSK9 target epitope titer at week 62 > 50 (expected to have PCSK9-specific serum antibody concentrations > 1 µg/ml), showed a decrease in LDLc values from baseline at week 70 and week 90 (Fig. 4c). In this group, the maximal individual LDLc decrease was 39% at week 90, suggesting that higher immunogenicity has a more favourable impact on lipid metabolism.

Although maximal antibody titers were observed at week 10 (study Part A) and week 62 (Part C; Fig. 2a), the maximal LDLc decrease was detected several weeks later in week 20 (Part A) and week 90 (Part C), respectively (Fig. 3a). In both active treatment groups, HDLc did not change over time (data not shown).

To understand the relationship between immunogenicity and effect on lipid parameters, a correlation analysis was performed. An inverse correlation between LDLc levels and immune response against AT04A was observed (especially after the booster) with an increasingly significant inverse correlation covering the period from week 66 (4 weeks after the peak titer at week 62) to week 82 with a Spearman’s correlation coefficient of up to *r* = − 0.51, supporting the theory that higher antibody concentrations lower LDLc.

## Discussion

The novel approach of a specific active immunotherapy targeting PCSK9 was assessed in this first-in-human study demonstrating the safety and tolerability of AT04A and AT06A. Injection site reactions represented the majority of AEs (63% of all AE), comprising erythema, induration, swelling, granuloma, and pain, which are commonly reported AEs in vaccination studies [21]. Most ISRs were mild or moderate. Severe ISRs were observed only in the active treatment groups; however, they were transient, rarely requiring short-term medication. The number of local reactions did not increase with priming immunizations; however, the severity of ISRs intensified after the booster. The increased intensity of ISRs after booster immunization might be associated with the higher booster dose used. Further dose finding studies will be required for clarification. With regard to systemic TEAEs, the safety profile of AT04A was comparable to that of placebo.

No similar therapeutic for hypercholesterolemia treatment has entered clinical trials yet, although two monoclonal antibodies to PCSK9 for passive immunotherapy have received market authorization.

The AE profile in our study was comparable to that reported for monoclonal antibodies, where ISRs included pain and erythema without safety issues in liver or muscle [13, 14, 22].

Mounting an immunological response against self-antigens is challenging, as immunological tolerance must be overcome, without inducing harmful autoimmune responses. Early clinical trials with Alzheimer’s disease candidate vaccine (AN1792) utilized the entire amyloid-β peptide as an immunogen, which also contained T-cell epitopes and immunization resulted in T cell-mediated microencephalitis cases [23]. In our study, PBMCs isolated from all enrolled subjects before and after treatment were investigated for the presence of PCSK9-target-epitope-specific T cells by ELISPOT, with no target sequence-specific T cell activation following exposure to either active treatment, confirming the safety and tolerability of SAIT. Off-target reactivity of antibodies was not assessed experimentally; however, clinical safety data gave no signal for off-target reactivity of treatment-induced antibodies. Furthermore, AT04-induced antibodies did not show cross-reactivity to other circulating proteins in preclinical studies [19].

Immunizations with both AFFITOPE® conjugates induced a long-lasting IgG antibody response against the immunizing peptides at a dose of 15 µg, which also resulted in high cross-reactivity to the native PCSK9 target epitope. The immune responses were strongly reactivated following booster immunization with 75 µg peptide, with seroconversion in 88% of subjects on active treatment, consistent with stimulation of PCSK9-specific memory B cells.

AFFITOPE® treatment did not result in a significant change of group mean total or antibody-free PCSK9 protein levels over time compared to placebo. In evolocumab clinical studies, a group mean serum antibody concentration in the low single-digit µg/mL (about 2–3 µg/mL) range was designated a threshold for modulation of serum PCSK9 protein levels [24]. As group mean antibody concentrations in both treatment groups were approximately at/below this level (around 1–2 µg/mL), a measurable change in serum PCSK9 protein levels may not be expected. However, over the study period, AT04A treatment resulted in a statistically significant decrease in LDLc compared to placebo (*P < 0.0001)*, and titers > 50 (indicative for PCSK9-specific antibody concentrations > 0.5–1 µg/ml) were associated with clinically relevant changes [25, 26] in LDLc (Fig. 4c), confirming preclinical study findings [19]. The clear correlation between AT04-induced antibodies and reduction of LDLc is unlikely to be associated with non-specific treatment-induced effects, such as local cytokine release upon injection, as treatment with AT06 and placebo did not reduce LDLc.

Given the long-lasting impact on LDLc levels, AT04A was chosen as the candidate for future development, aiming to enhance immunogenicity, while maintaining the favorable safety profile. Subjects with high titers showed a decrease in LDLc levels up to 39%, which lasted to the study end, with a trend toward further decreasing LDLc values (Supplementary Fig. 1). Thus, it is possible that the full potential of the lipid-lowering effect by SAIT may not have been fully assessed during this study period. AT06A induced similar titers to AT04A, but did not influence lipid parameters significantly. The PCSK9-mimicking sequences differ in both immunotherapeutics; presumably the induced antibodies recognize slightly different epitopes on the native circulating PCSK9 protein not manifested by ELISA.

An active immunotherapy approach offers potential advantages over monoclonal antibody therapy, namely long-lasting immunity, considerably lower cost and no need for frequent self-administration. The pharmacodynamic effects of AT04 immunization in healthy subjects demonstrated in this study are evidence for correct and sustained target engagement and warrant assessment of the clinical effects in patients with elevated LDLc and atherosclerotic disease. Further studies are planned using a formulation modified to enhance immunogenicity by use of the AT04 peptide sequence conjugated to a more powerful carrier protein.

## Conclusion

The specific active immunotherapies evaluated in this study were safe and induced a long-lasting immune response against the immunizing peptides, which translated into an immune response against the PCSK9 target epitope, resulting in significant lowering of LDLc levels following AT04A treatment over time. PCSK9 epitope titers correlated inversely with changes in LDLc levels, higher titers being associated with lower LDLc levels. The immune response was readily re-activated by a booster immunization, paving the way for patient-friendly therapeutic schedules. The safety profile and statistically significant effect on LDLc-lowering in this phase 1 trial justifies further clinical development of AT04 therapy.

## Supplementary Information

Below is the link to the electronic supplementary material.

Supplementary file1 (PDF 427 KB)
