# Supplementary material for: A phase I study assessing the safety, tolerability, immunogenicity, and low-density lipoprotein cholesterol-lowering activity of immunotherapeutics targeting PCSK9
Source: Eur J Clin Pharmacol. 2021 May 10;77(10):1473–84. doi: 10.1007/s00228-021-03149-2 (PMC8440313; doi:10.1007/s00228-021-03149-2)
Supplement: Supplementary file 1 — Supplementary file1 (PDF 427 KB) [file 228_2021_3149_MOESM1_ESM.pdf]

Supplementary Information

A phase I study assessing the safety, tolerability, immunogenicity and low-density lipoprotein cholesterol-lowering activity of immunotherapeutics targeting PCSK9

Category: Clinical Trial

Markus Zeitlinger, MD [1,*]; Martin Bauer, MD [1,*]; Roman Reindl-Schwaighofer, MD [1]; Robert M. Stoekenbroek, MD PhD [2]; Gilles Lambert, MD PhD[3]; Evelyn Berger-Sieczkowski, MD PhD [4]; Heimo Lagler, MD [5]; Zoe Oesterreicher, MD [1]; Beatrix Wulkersdorfer, MD [1]; Petra Lührs, PhD [6]; Gergana Galabova, PhD [6,7]; Carsten Schwenke, PhD [8]; Robert M. Mader, PhD [5]; Rossella Medori, MD [6]; Christine Landlinger, PhD [6]; Alexandra Kutzelnigg, MD [6]; Günther Staffler, PhD [6]

[1] Department of Clinical Pharmacology, Medical University of Vienna, Währinger Gürtel 18-20, 1090 Vienna, Austria

[2] Department of Vascular Surgery, Academic Medical Center, University of Amsterdam, P.O. Box 22660, 1100 DD Amsterdam, the Netherlands

[3] Laboratoire Inserm UMR 1188 DéTROI, Université de La Réunion, 2 Rue Maxime Rivière, 97490 Sainte Clotilde, France

[4] Department of Neurology, Medical University of Vienna, Währinger Gürtel 18-20, 1090 Vienna, Austria

[5] Department of Medicine I, Medical University of Vienna, Währinger Gürtel 18-20, 1090 Vienna, Austria

[6] AFFiRiS AG, Karl Farkas Gasse 22, 1030 Vienna, Austria

[7] Present address: Origenis GmbH Am Klopferspitz 19a, 82152 Martinsried, Germany

[8] SCO:SSiS, Karmeliterweg 42, 13465 Berlin, Germany

* Both authors contributed equally to the results of this study

Correspondence to:
Günther Staffler
AFFiRiS AG
Karl Farkas Gasse 22, 1030 Vienna, Austria
Tel.: +43 1 798 15 75 7002
Fax: +43 1 798 15 75 311
Email: guenther.staffler@affiris.com

supplementary Table 1: Serious Adverse Events overview

supplementary Table 2. Therapy-related systemic adverse events by treatment group

supplementary Table 3. Severe systemic TEAE by SOC

supplementary Table 4. Total PCSK9 blood levels over time by treatment group

supplementary Table 5. Free PCSK9 blood levels over time by treatment group

supplementary Table 6. Exploratory analysis of LDL cholesterol over time

supplementary Table 1: Serious Adverse Events overview

SOC Term	PT Term	AT04A	AT06A	Placebo	Total
Gastrointestinal disorders	Esophageal motility disorder	0	0	1	1
Injury, poisoning and procedural complications	Meniscus injury	1	0	1	2
	Tendon rupture	0	1	0	1
Nervous system disorders	Sciatica	0	0	1	1
Nervous system disorders	Carpal tunnel syndrome	1	0	0	1
Surgical and medical procedures	Removal of foreign body from gastrointestinal tract	0	0	1	1
Sum		2	1	4	7

Legend: Absolute numbers of experienced SAE coded according to MedDRA

supplementary Table 2. Therapy-related systemic adverse events by treatment group

Severity	AT04A	AT06A	Placebo	Total
Mild	25	60	43	128
Moderate	9	10	4	23
Severe	0	1*	0	1
Total	34	71	47	152

Legend: Numbers represent the number of systemic AE classified as treatment-related

* Subject 073 (treatment: AFFITOPE® AT06A conjugate) experienced a severe systemic TEAE, a transient episode of asthma, considered probably related to treatment. The subject had no previous history of asthma, and recovered within one day supported by medication (inhalation of fenoterol/ipratropium bromide). The event was discussed in the DSMB and did not raise safety concerns, however, as a preventive action, no third immunization was administered.

supplementary Table 3. Severe systemic TEAE by SOC

SOC	AT04A	AT06A	Placebo	Total
General disorders and administration site conditions	3	1	2	6
Infections and infestation	6	1	3	10
Injury, poisoning and procedural complications	2	3	2	7
Metabolism and nutrition disorders	0	1	0	1
Musculoskeletal and connective tissue disorders	1	0	1	2
Nervous system disorders	3	0	3	6
Psychiatric disorders	2	0	0	2
Renal and urinary disorders	0	0	1	1
Reproductive system and breast disorders	2	0	0	2
Respiratory, thoracic and mediastinal disorders	0	2	1	3
Sum	19	8	13	40

Legend: TEAE = treatment-emergent adverse event

supplementary Table 4. Total PCSK9 blood levels over time by treatment group

Legend: n = number of subjects

Timepoint	Parameter	Total PCSK9 in Blood [ng/ml]		
		AT04A	AT06A	Placebo
Week 0 – Visit 2	Mean	330.29	320.67	342.42
	Range	203-490	199-530	205-592
	n	24	24	24
Week 10 – Visit 7	Mean	336.96	323.75	346.67
	Range	222-603	193-469	246-596
	n	24	25	24
Week 52 – Visit 10	Mean	295.65	295.87	297.42
	Range	185-434	177-538	169-432
	n	23	23	24
Week 60 – Visit 11	Mean	307.73	283.18	309.61
	Range	218-493	164-451	219-461
	n	15	17	18
Week 66 – Visit 13	Mean	291.80	295.06	314.83
	Range	182-401	184-397	197-415
	n	15	16	18
Week 90 – Visit 17	Mean	294.21	276.69	294.39
	Range	190-607	162-419	203-408
	n	14	16	18

supplementary Table 5. Free PCSK9 blood levels over time by treatment group

Timepoint	Parameter	Free PCSK9 in Blood [ng/ml]		
		AT04A	AT06A	Placebo
Week 0 – Visit 2	Mean	253.79	261.21	273.00
	Range	84-419	80-445	80-652
	n	24	24	24
Week 10 – Visit 7	Mean	296.54	269.46	263.25
	Range	145-695	91-530	119-540
	n	24	24	24
Week 52 – Visit 10	Mean	161.22	159.09	194.00
	Range	58-505	66-420	62-467
	n	23	23	24
Week 60 – Visit 11	Mean	159.60	164.47	173.39
	Range	63-316	71-304	82-381
	n	15	17	18
Week 66 – Visit 13	Mean	159.87	156.44	162.44
	Range	74-266	52-293	67-244
	n	15	16	18
Week 90 – Visit 17	Mean	142.29	146.25	140.61
	Range	51-348	45-285	57-277
	n	14	16	18

Legend: n = number of subjects

supplementary Table 6. Exploratory analysis of LDL cholesterol over time

Comparison	All visits	
	**Mean difference [95% CI]**	**P value**
AT04A-AT06A	-6.9 [-10.3; -3.5]	0.0002
AT04A-Placebo	-7.2 [-10.4; -3.9]	<0.0001
AT06A-Placebo	-0.3 [-3.4; 2.8]	0.8509

Legend: The mean difference is the average of LDLc differences between the groups' relative changes over time with baseline as cofactor in the statistical model; CI = confidence interval
